# scaRNA1 Expression Levels Affect Alternative Splicing of mRNA

**DOI:** 10.3390/genes16080864

**Published:** 2025-07-24

**Authors:** Madeleine Brown, Brittnei Earl, Michael Filla, Nataliya Kibiryeva, James E. O’Brien, Douglas C. Bittel

**Affiliations:** 1 College of Biosciences, Kansas City University, Kansas City, MO 64106, USA; reims98@gmail.com (M.B.); brittnei.earl@kansascity.edu (B.E.); nkibiryeva@kansascity.edu (N.K.); 2 Ward Family Heart Center, Children’s Mercy Hospital, Kansas City, MO 64108, USA; jobrien@cmh.edu

**Keywords:** epigenetics, spliceosome, transcriptome, alternative splicing

## Abstract

Our previous research identified 12 small Cajal body-specific RNAs (scaRNAs) with reduced expression in the right ventricle in infant patients with tetralogy of Fallot. Likewise, we showed that there were significant changes in mRNA processing in the RV in these patients. ScaRNAs play a crucial role in the biochemical maturation of spliceosomal RNAs (pseudouridylation and 2′-O-methylation). We showed that variations in scaRNA1 levels resulted in changes in alternative splicing in human cells. To investigate further the role that scaRNAs play in mRNA processing, we examine here the impact of knocking down scaRNA1 in quail myoblast cells (*Coturnix japonica*, a well-established animal model for studying embryonic development). Following the knockdown of scaRNA1, transcriptome analysis revealed that the genes *Tjp1*, *Map3k7*, and *Sppl2a* were alternatively spliced. Growing evidence indicates that alternative splicing of mRNA plays an important role in regulating cell differentiation and tissue development. Our data presented here provide additional support for research to clarify the specific roles that individual scaRNAs play in regulating spliceosome function and mRNA splicing.

## 1. Introduction

Small Cajal body-specific RNAs (scaRNAs) play a crucial role in the biochemical maturation of spliceosomal RNAs (pseudouridylation and 2′-O-methylation). Our previous research established an association between insufficient levels of scaRNA expression and changes in mRNA processing (alternative splicing) [1,2]. We further showed dysregulation of scaRNA levels and mRNA splicing in the right ventricles of infants with tetralogy of Fallot (TOF) [3]. In human cell cultures, we showed that variations in scaRNA levels result in changes in pseudouridylation and alternative splicing [3]. We hypothesize that scaRNAs are vital for spliceosomal function, and help determine mRNA splicing patterns as cells differentiate.

In the developing fetus, tissue differentiation and organ formation require complex coordination among interconnected signaling pathways [4]. There is growing evidence that proper mRNA splicing is crucial for organogenesis, including cardiogenesis [3,5,6,7,8,9]. During development, intricate and tightly regulated changes in gene expression and mRNA splicing lead to generation of the complex proteome necessary for establishing the structures and functions of tissues and organs. This includes the appropriate mRNA isoforms and corresponding protein isoforms of important transcription factors, signaling molecules, and structural proteins. Aberrations in pre-mRNA splicing during development may contribute to developmental defects [3]. Thus, scaRNAs may play a regulatory role through their involvement in the precise biochemical modification of spliceosomal RNAs (snRNAs) that are essential components of the spliceosome.

Previously, we characterized transcriptomes extracted from the right ventricle (RV) in infants with tetralogy of Fallot (TOF) [7]. We found that 12 scaRNAs were significantly downregulated in the RV in infants with TOF, compared to normally developing infants. Through their participation in the modification and maturation of snRNAs, scaRNAs help ensure the precise splicing of pre-mRNA, a critical process for generating mature mRNA and the proteome. One of the 12 downregulated scaRNAs, scaRNA1, pseudouridylates uridine 89 (referred to as Ψ^89^) of snU2. ScaRNA1 is conserved in fish, birds, and mammals, suggesting that Ψ^89^ is important for the function of the U2 subunit in vertebrates. We demonstrated that a decrease in scaRNA1 expression leads to reduced levels of U2 Ψ^89^ and subsequent changes in mRNA splicing in human cells. Our studies of zebrafish showed that changes in scaRNA expression levels cause changes in mRNA isoform expression, resulting in dysregulation of heart development [3]. Similarly, mice lacking the pre-mRNA regulator QKI exhibit dysregulation of alternatively spliced genes involved in cardiac contractile physiology and myofibrillogenesis [10].

Here, using quail myoblasts, we present evidence that scaRNA1 knockdown (KD) impacts alternative splicing in a third species. Bird embryos have been used for over 100 years to study vertebrate cardiac development. Our current work indicates that scaRNA function is conserved in quail. Quail embryos should therefore be a useful model in studies of the molecular genetics of scaRNA impact on the function of the spliceosome and, in turn, in regulating development. Using transcriptome analysis of high-resolution RNA-Seq data, we identified multiple cardiogenesis-promoting genes that exhibit significant alternative splicing events after scaRNA1 knockdown. Taken together, these findings highlight the importance of scaRNAs in the fundamental processes of mRNA splicing and, potentially, in regulating development.

## 2. Methods

### 2.1. QM7 Cell Culture

QM7 cells (ATCC, Manassas, VA, USA) were grown in M199 complete media that contained 80% 1× M199 media (Gibco, Billings, MT, USA), 10% fetal bovine serum (Gibco, Billings, MT, USA), 10% 1× tryptose phosphate broth (Gibco, Billings, MT, USA), and 1% each of 100× Pen-Strep (VWR LifeScience, Randor, PA, USA), 100 mM Na pyruvate (Gibco, Billings, MT, USA), and 100× GlutaMax (Gibco, Billings, MT, USA). QM7 cells were seeded into 6-well tissue culture plates at a density of 2.0 × 10^5^ cells per well in 2 mL complete media and allowed to grow overnight. Cells were transfected using Lipofectamine 3000 (Thermo Fisher Scientific, Waltham, MA, USA, L3000008) according to the manufacturer’s instructions. To each well, 20 pmols of control (scrambled non-targeted) or scaRNA1-targeted CAO was added after performing a media change. Cultures were incubated a 37 °C in 5.0% CO_2_ humidified incubator for 48 h.

### 2.2. Extracting and Quantifying RNA

RNA was extracted following the Phasemaker Tube protocol (Thermo Fisher Scientific, Waltham, MA, USA, A33248). The extracted RNA was quantified using a NanoDrop 2000 spectrophotometer (ThermoFisher, Waltham, MA, USA) and stored at −80 °C for future use.

### 2.3. DNaseI Treatment

RNA samples of 10 µg weight were purified using DNase-free kit (Thermo Fisher Scientific, Waltham, MA, USA, AM1906) in accordance with the manufacturer’s protocols. Following addition of the DNase Removal Resin, samples were mixed and spun for 30 s at 13,000× *g* three times to encourage maximum efficiency of the DNase Removal Resin. Purified samples were used immediately or stored at −80 °C.

### 2.4. Reverse Transcriptase (RT)

Using the SuperScript IV First-Strand Synthesis System (Thermo Fisher Scientific, Waltham, MA, USA, 01236186), 0.5 ug of treated RNA was reverse-transcribed following the manufacturer’s protocol using oligo dTs. The reaction mix was incubated at 23 °C for 10 min, followed by 50 °C for 10 min. The reaction was then inactivated by incubating at 80 °C for 10 min. The cDNA product was used immediately or stored at −20 °C for future use.

### 2.5. Quantitative Polymerase Chain Reaction (qPCR)

Quantitative PCR was performed using Power SYBR Green PCR MasterMix (Thermo Fisher Scientific, Waltham, MA, USA, 4367659), following the manufacturer’s protocol, on a CFX96 Touch Real-Time PCR Detection System (Bio Rad, Hercules, CA, USA). The protocol was as follows: (Stage 1) hot start 95 °C for 10 min; (Stage 2) 40 cycles of 95 °C for 10 s, 60 °C for 30 s, 72 °C for 45 s; (Stage 3) generation of a melt curve by heating the annealed amplicons from 60 °C to 95 °C, reading at 0.5 °C increments. This procedure was carried out three times for each sample. Primers for qPCR assessment of scaRNA1 and Ppp1r8 (host gene) after knockdown of scaRNA1 are shown in Table 1.

### 2.6. Isoform-Specific Primers

**Figure 1:**
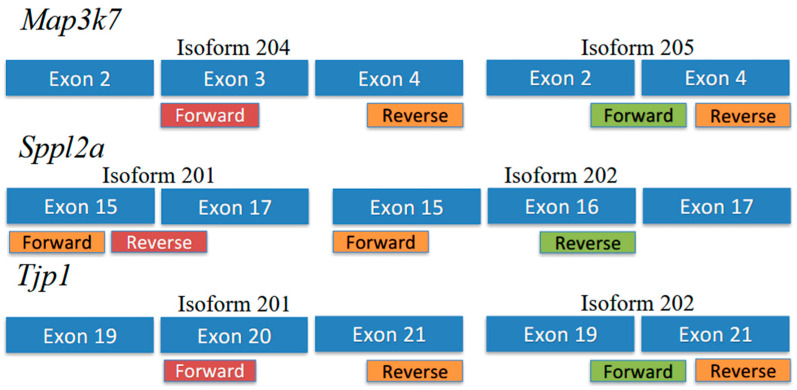
Isoform-specific primer positions and sequences for qPCR validation of RNA-seq results (see Table 2 for primer sequences).

Primer sequences are shown in Table 2. For *Tjp1* and *Map3k7*, primers were designed so that both isoforms shared a reverse primer, and each isoform had a specific forward primer (Figure 1). For *Sppl2a*, primers were designed so that both isoforms shared a forward primer, and each isoform had a specific reverse primer (Figure 1).

### 2.7. CMC Treatment and Quantification of Pseudouridylation (Ψ)

**Figure 2:**
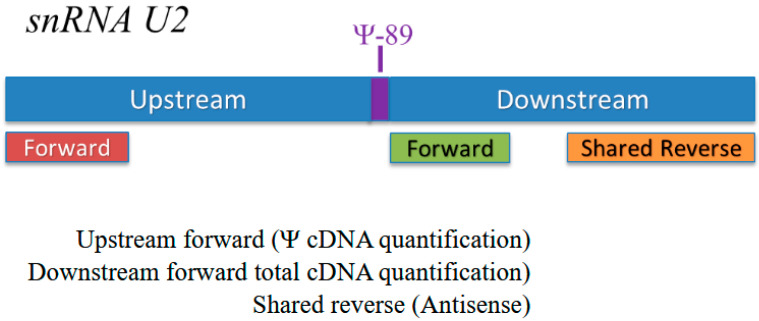
Positions of primers used by CMC-Seq to measure pseudouridylation levels at Ψ-89.

CMC [N-cyclohexyl-N′-β-(4-methylmorpholinium) ethyl carbodiimide] (Santa Cruz Biotechnology, CAT# CAS2491-17–0) treatment was performed on previously extracted RNA as described by Nagasawa et al. [2]. Quantification of Ψ^89^ is described in detail in [2]. Briefly, RNA samples that are pseudouridylated at Ψ^89^ in snRNA U2 will acquire a bulky side chain at that position post CMC treatment that causes reverse transcription to stall at Ψ^89^. This results in the reduction of the long amplicon after CMC treatment, compared to the non-CMC-treated RNA. Thus, there is an inverse relationship between the loss of the long amplicon and the amount of Ψ^89^. There is a minor reduction in the efficiency of reverse transcription post CMC treatment. The short amplicon is not impacted by CMC treatment; therefore, the quantity of the short amplicon can be used to normalize between CMC treatment and control (Figure 2). We determined the amount of Ψ by calculating the ratio (R) between the relative change in the long amplicon before and after CMC treatment and the relative change in the short amplicon before and after CMC treatment. The equation is a semi-quantitative representation of the differences between multiple samples. The equation is as follows: R = [(Elong amplicon) ΔCt long amplicon (MEAN BEU − MEAN CMC)]/[(Eshort amplicon) ΔCt short amplicon (MEAN BEU − MEAN CMC)], where R represents ratio, E represents the assay efficiency, and ΔCt represents the differences between the averages of the non-CMC-treated samples and the CMC-treated samples. Sequences of primers used by CMC-Seq to quantify Ψ-89 are shown in Table 3.

### 2.8. RNA-Seq

RNA-seq was performed at the Genomic Medicine Center (Children’s Mercy Hospital, Kansas City, MO, USA) using the TruSeq Stranded Total RNA Sample Prep Kit (# RS-122-2201, Illumina, San Diego, CA, USA). Sequencing was conducted in high-output run mode on the HiSeq1500 platform, following a previously published protocol.

Each sample was sequenced using 2 × 101 bp paired-end reads to an average depth of ~6 billion reads, with >86% of bases exceeding Q30 quality scores. Adapter sequences, rRNA contamination, and poly-A tails were removed before alignment. The resulting base calling (.bcl) files were converted to FASTQ format using Illumina’s bcl2fastq v2.17.1.14 software.

### 2.9. RNA-Seq Bioinformatic Analysis

FASTQ files were uploaded to PartekFLOW 12.4.3 (Illumina) for processing. Pre-alignment QC/QA was performed to assess raw sequencing read quality, and low-quality reads were trimmed using a Phred quality score threshold of >30 with a minimum read length of 25 bp (the algorithm is built into PartekFLOW). Reads were aligned to the Homo Sapience reference transcriptome (hg38) using the splice-aware aligner (STAR) 2.7.8a. Transcript- and gene-level summaries were generated based on Ensembl transcripts (release 112). During alignment we filtered out reads that mapped more than 10 locations, had more than 10 mismatches, or had a mismatch-to-mapped ratio > 0.3. Post alignment, duplicates, low-quality reads (mapping quality < 20), singletons, and unaligned reads were removed to ensure high-quality data. Aligned reads were then quantified using the Ensembl (release 112) annotation model with requirement of strict paired-end compatibility and a minimum of 95% read length overlap with the annotated feature. Only features with 10 or more reads across all samples were included in subsequent analysis which was carried out to explore splicing isoforms (transcript-level).

To assess splicing isoforms (transcript-level), we used Transcripts Per Million (TPM) normalization (allowing comparison of RNA transcript expression within a single sample) and included only protein-coding genes for downstream analysis. Gene-specific ANOVA was used to detect alternative splicing events, with an Alt-splice index range of 2 to 100 and significance threshold *p* ≤ 0.05 after Bonferroni correction for multiple testing.

## 3. Results

**Figure 3:**
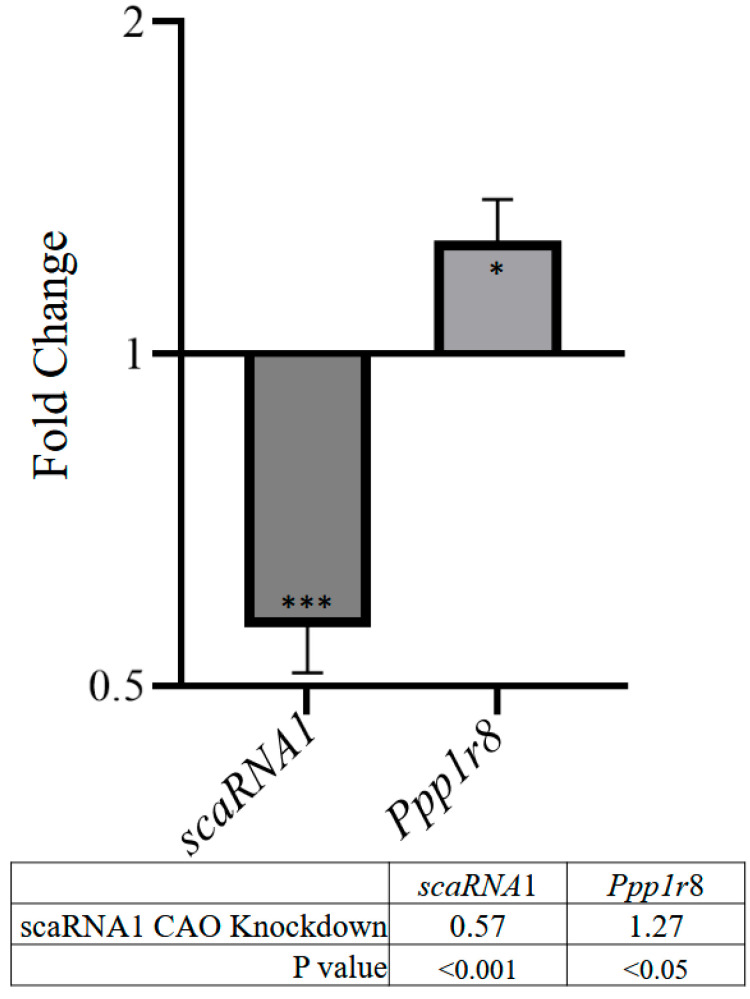
Analysis of scaRNA1 knockdown and Ppp1r8 host gene by qPCR, comparing cells transfected with a non-targeted scramble and an scaRNA1-targeted chimeric antisense oligonucleotide (CAO). n = 9. * *p* < 0.05. *** *p* <0.001.

**Figure 4:**
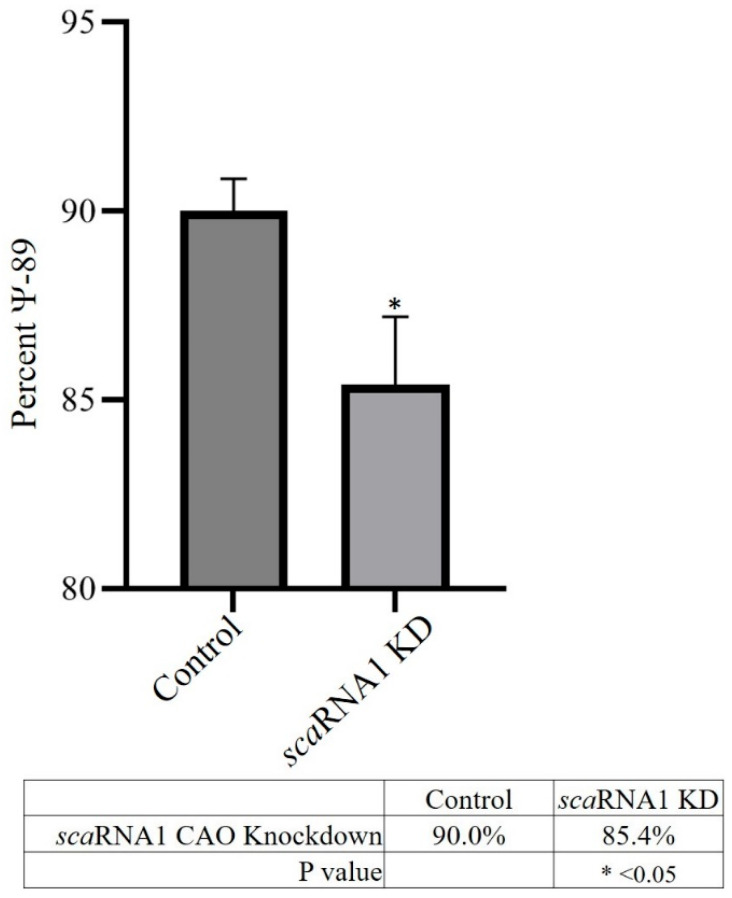
Percentages of Ψ-89 in non-targeted scramble control and *sca*RNA1 targeted for knockdown by CAO. * *p* < 0.05.

Knockdown (KD) of scaRNA1 in quail QM7 cells was accomplished using chimeric antisense oligonucleotides (CAOs) designed to target the scaRNA1 transcript. The levels of scaRNA1 were analyzed using quantitative PCR (qPCR). Figure 3 shows that, on average, scaRNA1 exhibited a nearly 2-fold decrease in expression when targeted with CAOs. Pseudouridylation analysis (Figure 4) showed that there was a 5% decrease in Ψ^89^ in scaRNA1 KD samples.

We identified 23,371 transcripts in the quail genome. Of these, 14,008 had an AS splicing index between 2 and 100. However, after applying Bonferroni correction for multiple testing, only three genes were identified as alternatively spliced—*Tjp1*, *Map3k7*, and *Sppl2a*. We focused on these three genes for qPCR validation. Figure 1 shows the positions of isoform-specific primers used to amplify isoform transcripts for each gene.

**Figure 5:**
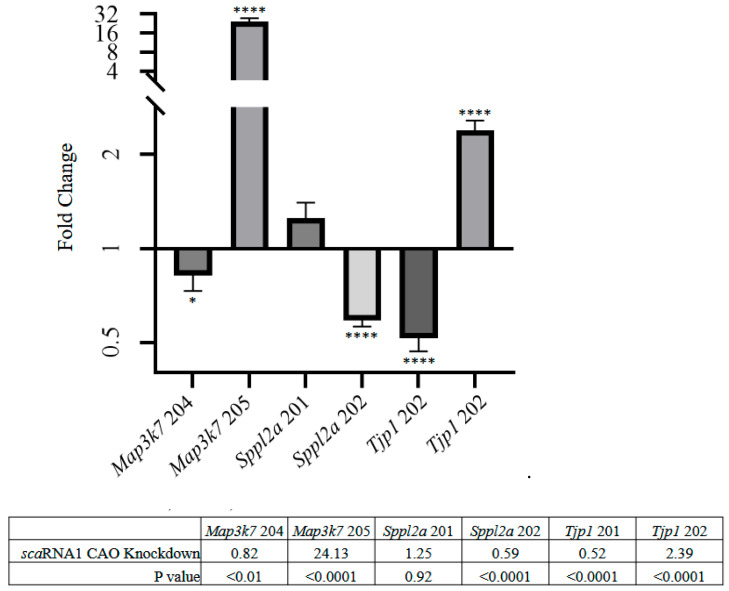
mRNA isoform-specific qPCR analysis in QM7 cells after Scarna1 knockdown by CAOs. * *p* < 0.01, **** *p* < 0.0001.

Figure 5 shows that *Tjp1 isoform 201* exhibited a nearly 2-fold decrease, *isoform 202* a 2.4-fold increase, *Map3k7 isoform 205* a 24-fold increase, and *Sppl2a isoform 202* a nearly 2-fold decrease.

## 4. Discussion

Interest in the function of alternatively spliced messenger RNA (mRNA) is rapidly growing, underscoring the need for deeper research into the mechanisms that regulate pre-mRNA processing. Alternative splicing is a crucial process that allows a single gene to produce multiple protein isoforms, thereby increasing the diversity of the proteome. Our study investigates the role of small Cajal body-specific RNAs (scaRNAs) in this process, particularly focusing on their influence on the biochemical modification of spliceosomal RNAs. We hypothesize that scaRNAs, specifically scaRNA1, play a significant role in mRNA alternative splicing and that this may have implications for cell differentiation and organ development. Our findings suggest that changes in scaRNA1 expression can alter the splicing patterns of mRNAs, potentially impacting the function of genes critical for regulating development. With high statistical stringency, our analysis identified three genes that are alternatively spliced after scaRNA1 knockdown: Tjp1 (tight junction protein 1); Map3k7 (mitogen-activated protein kinase kinase kinase 7); and Sppl2a (signal peptide peptidase-like 2A).

Tjp1 is a key regulator of cell migration, and is vital for fetal survival [11]. Mice deficient in Tjp1 die after about 10 days of gestation with notable embryonic and extraembryonic abnormalities. Embryos lacking Tjp1 exhibit neural tube and notochord defects, as well as allantois disorganization. Furthermore, Tjp1-deficient mice show impaired angiogenesis in the yolk sac. In addition to these key developmental functions, Tjp1 regulates paracellular permeability and interacts with various transmembrane proteins and the actin cytoskeleton [12]. Importantly, alternative splicing of Tjp1, resulting in the absence of exon 20, leads to altered endothelial permeability, which is evident both in vitro and in vivo [13]. This alteration causes increased immune-cell infiltration in the heart, eventually leading to myocarditis or other cardiac pathologies. Reduced survival was noted in mice lacking exon 20 of Tjp1 compared to control mice.

Splicing variant analysis of Map3k7 revealed that, when scaRNA1 is knocked down, Map3k7-205 shows a more-than-24-fold increase in expression relative to samples without scaRNA1 knockdown. In wild-type samples, Map3k7-205 was not detected by qPCR, which may explain the large increase in the isoform’s presence when scaRNA1 is knocked down. Map3k7-205 is the splice variant lacking exon 3, which contains the Tab1 binding domain. Disruption of Tab1 function has been shown to be fatal in mouse models [14,15]. Downstream dysfunction is likely due to the loss of the Tab1 binding domain, potentially leading to cardiac and lung dysmorphogenesis and embryonic lethality.

Analysis of Sppl2a revealed that, when scaRNA1 is knocked down, Sppl2a-202 shows a nearly 2-fold decrease in expression. Sppl2a-202 is the variant containing exon 16, the presence or absence of which is of unknown importance. Sppl2a has implications in cardiac development, as it helps control cellular levels of VAMP 1-4 (vesicle-associated membrane protein) by initiating their degradation, possibly impacting cellular trafficking [16]. VAMP-1 and VAMP-2 form a SNARE complex inside cardiac myocytes that helps release and regulate the amount of atrial natriuretic peptide (ANP), the lack of which leads to ventricular hypertrophy [17,18].

Small Cajal body-specific RNAs play an essential role in the biogenesis and function of spliceosomal RNAs. Thus, scaRNAs help ensure the precise splicing of pre-mRNA, a process critical for the generation of mature mRNA, regulation of gene expression, and addition of variation to the proteome. Here, we showed that the level of scaRNA1 is directly related to the level of Ψ^89^ and leading to changes in mRNA splicing of three genes that are important for regulating heart development. The role that alternative mRNA splicing plays in regulating development remains unclear. Previous research by our laboratory revealed that infants born with tetralogy of Fallot had decreased levels of twelve small Cajal body-specific RNAs along with altered mRNA splicing of genes important for embryonic development. Taken together, these observations suggest that the scaRNA level regulates biochemical modifications of spliceosomal RNAs that influence alternative splicing of mRNAs which may play a role in cell differentiation. Clearly, there is a need for further research to explore the biological repercussions of altered post-transcriptional modifications of spliceosomal RNAs and the role that scaRNAs play in regulating alternative splicing and, ultimately, in regulating vertebrate development.

## Figures and Tables

**Table 1:** Primers for qPCR assessment of scaRNA1 and Ppp1r8 (host gene) after knockdown of scaRNA1.

Primer Name	Sequence
*scaRNA1* forward	AGCACTTGATACTAACTGAGCTATC
*scaRNA1* reverse (antisense)	TCTGAGCTATAACCCAGTGTCA
*Ppp1r8* forward	AAAACCTCCACCTGGCTTACA
*Ppp1r8* reverse (antisense)	TGTGATAGACCAAGGCAGCAT

**Table 2:** Isoform Specific Primers for qPCR.

Primer Name	Sequence
*Map*3*k*7-204 forward	TCGACAACTGTCACGAGTAAAC
*Map*3*k*7-205 forward	TTCATTGTAGAGGTGTGTCTTGT
*Map*3*k*7-205 shared reverse (antisense)	CACTCCTTGGGAACACTGTAAA
*Sppl*2*a* shared forward	CATTGGTATGGTGCTGACTTT
*Sppl*2*a*-201 reverse (antisense)	GGTAATCCAGATGTTCCATCAT
*Sppl*2*a*-202 reverse (antisense)	TTGTAGCAAAGGCGTCCTG
*Tjp*1-201 forward	GGGAGCTCATGTAGTGCTAAG
*Tjp*1-202 forward	CAGTACAGACAGCAGACATACAT
*Tjp*1 shared reverse (antisense)	GCCTCTGTACTGGGTGATTAAC

**Table 3:** Primers used for CMC-Seq to quantify Ψ-89.

Primer Name	Sequence
*snRNA U2* Upstream forward (Ψ cDNA quantification)	CTGATACGTCCTCGATGAGA
*snRNA U2* Downstream forward total cDNA quantification)	GTTGGACCCGGAGCTTGCTCCCTCCGCT
*snRNA U2* Shared reverse (Antisense)	TACTGCCATACCGGGACGATGCGC

## Data Availability

The original contributions presented in this study are included in the article. Further inquiries can be directed to the corresponding author.
